# In Vitro Digestibility and Bioaccessibility of Nutrients and Non-Nutrients Composing Extruded Brewers’ Spent Grain

**DOI:** 10.3390/nu14173480

**Published:** 2022-08-24

**Authors:** Maria Belen Gutierrez-Barrutia, Sonia Cozzano, Patricia Arcia, Maria Dolores del Castillo

**Affiliations:** 1 Departamento de Ingeniería, Universidad Católica del Uruguay, Montevideo 11600, Uruguay; 2 Instituto de Investigacion en Ciencias de la Alimentacion, CSIC-UAM, Nicolas Cabrera 9, 28049 Madrid, Spain; 3 Latitud-LATU Foundation, Av. Italia 6201, Montevideo 11500, Uruguay

**Keywords:** amino acids, anti-inflammatory, antioxidant, brewers’ spent grain, bioaccessibility, bioactive compounds, diabetes, extrusion, glucose transporters, short-chain fatty acids

## Abstract

This study aimed to evaluate the effect of the extrusion process on the bioaccessibility of brewers’ spent grain (BSG) nutrients (carbohydrates and proteins) and non-nutrients (bioactive compounds). BSG and extruded BSG (EBSG) were digested in vitro simulating human oral-gastro-intestinal digestion and colonic fermentation. The duodenal bioaccessibility of glucose, amino acids and phenolic compounds was analyzed. The fermentability of the dietary fiber was assessed by analysis of short-chain fatty acids. Additionally, assessment of the bioaccessibility of phenolic compounds after colonic fermentation was undertaken. The antioxidant, anti-inflammatory and antidiabetic properties of the bioaccessible compounds were studied. Extrusion caused no change in the digestibility of gluten and glucose bioaccessibility (*p* > 0.05). Moreover, the bioaccessibility of amino acids and phenolic compounds significantly increased (*p* < 0.05) due to extrusion. However, higher short-chain fatty acid content was formed in colonic fermentation of BSG (*p* < 0.05) compared to EBSG. The latter inhibited intracellular ROS formation in IEC-6 cells and showed anti-inflammatory properties in RAW264.7 cells. With respect to antidiabetic properties, glucose absorption was lower, and the inhibition of carbohydrases higher (*p* < 0.05), in the presence of EBSG compared to BSG. The effects of EBSG and BSG digests on glucose transporters were not significantly different (*p* > 0.05). In conclusion, extrusion positively affected the nutritional value and health-promoting properties of BSG.

## 1. Introduction

In line with the seventeen Sustainable Development Goals of the United Nations [1], food system transformation is required involving a transition towards more sustainable and healthy diets to ensure food and nutrition security for all. Food systems are being challenged by malnutrition, environmental changes, and resource scarcity, and their resilience to emergencies, such as pandemics, climate change and geopolitical forces, is essential [2]. Additionally, noncommunicable diseases (NCDs) account for 41 million deaths each year with 77% of all NCD deaths occurring in low- and middle-income countries [3]. There is a clear relationship between food insecurity and NCDs related to nutrition. Therefore, it is essential for everyone concerned to move towards sustainable and healthy diets worldwide.

Brewers’ spent grain (BSG) is the most abundant byproduct of the brewing industry. Its major components are dietary fiber (50%) and proteins (30%) [4], two macronutrients in very high demand by consumers and which are essential for achieving nutrition security. In addition, BSG is a valuable source of non-nutrients, including bioactive compounds, such as hydroxycinnamic acids [4]. Therefore, BSG has gained attention and is starting to be commercialized for human consumption in different forms [5,6,7].

A previous study has raised the possibility of the use of extruded BSG as a safe, healthy and sustainable ingredient in the human diet [8]. Extrusion is a thermomechanical process which applies high temperature and pressure over a short period of time [9]. It was found that, after extrusion, the soluble dietary fiber and extractable phenolic content of BSG was increased, while safe microbial standards were achieved, and no processing contaminants (acrylamide) were formed. Moreover, extruded BSG showed low free glucose content and protein quality parameters similar to those recorded for eggs, milk and soy [8].

Further studies which evaluate the effect of the processing of brewers’ spent grain and its incorporation into the food matrix might have on the digestibility and bioaccessibility of nutrients and bioactive compounds are needed. It has been reported that extrusion may improve protein digestibility and cause the release of bound phenolics [10,11,12], favoring their bioaccessibility in the gastrointestinal tract. Nevertheless, the structure and properties of the cell wall may be unaltered by mastication and digestion, affecting the bioaccessibility of macronutrients from plants [13]. Furthermore, the bioaccessibility of polyphenols may be reduced due to dietary fiber binding [14]. When indigested dietary fiber from the small intestine reaches the colon it can be fermented by microbiota, producing short-chain fatty acids [13]. Moreover, microbial enzymes help to release native polyphenols from the food matrix, which is an essential mechanism for them to pass through the intestinal barrier [15].

It is important to establish whether BSG’s bioaccessible compounds can exert health-promoting properties. In previous studies, the intestinal bioaccessibility of phenolic compounds from other cereal fractions with anti-inflammatory and antioxidant properties was reported [16,17]. These properties are important in preventing chronic NCDs involving energetic metabolism, such as diabetes [18]. Short-chain fatty acids can also regulate the characteristics of metabolic syndrome and modulate immune response to prevent colorectal cancer [19].

This study aimed to evaluate the duodenal and colonic bioaccessibility of nutrients (glucose and amino acids) and non-nutrients (phenolic compounds) derived from BSG and extruded BSG, and their health-promoting potential in key physiological processes associated with the development of NCDs, such as antioxidant, anti-inflammatory, and antidiabetic effects. The findings provide useful information for understanding the feasibility of using extruded BSG (EBSG) as an ingredient for achieving global nutrition security.

## 2. Materials and Methods

### 2.1. Materials

The chemicals used were of reagent grade. Digestive enzymes (α-amylase from human saliva-A0521, porcine gastric pepsin-P6887, porcine pancreatin-P1625, bile porcine extract-B8631), Folin reagent, 2,20-azinobis-(3-ethylbenzothiazoline-6-sulfonic acid) diammonium salt (ABTS), fluorescein (FL) disodium salt, 2,20-azobis (2-methylpropionamidine) dihydrochloride (AAPH), O-phtalaldehyde, acarbose, p-nitrophenyl-α-d-glucopyranoside and rat intestine acetone powder were purchased from Sigma-Aldrich (St. Louis, MO, USA). The glucose oxidase-peroxidase kit used was obtained from Spinreact (Girona, Spain). Quinine sulphate dihydrated, p-nitrophenyl and a Pierce^®®^ Microplate BCA Protein Assay Kit—Reducing Agent Compatible were bought from Thermo Scientific™ (Waltham, MA, USA). Ferulic acid was obtained from Fluka Honeywell, Buchs, Switzerland.

For cellular studies, Dulbecco’s Modified Eagle’s Medium (DMEM), L-glutamine, antibiotics (penicillin and streptomycin) and trypsin were obtained from Gibco Laboratory (Invitrogen Co., Grand Island, NY, USA) and fetal bovine serum (FBS) was obtained from Hyclone (GE Healthcare, Chicago, IL, USA). 3-(4,5-dimethylthiazol-2-yl)-2,5-diphenyltetrazolium bromine (MTT), 2,7-dichlorofluorescin diacetate (DCFH-DA), sulfanilamide, *N*-1-(naphthyl)ethylenediamine-dihydrochloride, phosphoric acid, sodium nitrite and lipopolysaccharide from E. coli O55:B5 (LPS) were purchased from Sigma-Aldrich (St. Louis, MO, USA). Glucose transport inhibition standards (phloretin ≥ 99% and phloridzin dihydrate 99%) were purchased from Sigma-Aldrich (St. Louis, MO, USA).

### 2.2. Food Ingredient

Brewers’ spent grain (BSG) was obtained from Fábricas Nacionales de Cerveza (Minas, Uruguay) from a pool of one day’s production of lager beer. It was stabilized by drying in a convection oven (Atmos Promat 1, Alfa-Laval Gruppe, Glinde, Germany) at 45 °C ± 2 °C. Then, it was ground in a laboratory mill (Retsch ZM 200, Thermo Fisher Scientific™, Waltham, MA, USA) until its particle size was 0.5 mm.

Extruded BSG (EBSG) was obtained using a single screw extruder (Brabender Co Cordero E330, Brabender, Duisburg, Germany) with a die diameter of 4 mm, screw diameter of 1.8 cm and screw length equal to 40 cm. The thermomechanical processing conditions were 15.8% of sample moisture content, 164.3 revolutions per minute (rpm) screw speed and 122.5 °C barrel temperature, while the feeding rate was kept constant (100 rpm) [8]. Three series of extrusion processes were performed to obtain a pool of EBSG from different days, which were ground to 0.5 mm and kept at −20 °C for further analysis.

### 2.3. Duodenal Bioaccessibility of Nutrients and Non-Nutrients

#### 2.3.1. In Vitro Oral-Gastro-Intestinal Digestion

BSG and extruded brewers’ spent grain (EBSG) were subjected to in vitro oral-gastro-intestinal digestion according to Hollebeeck et al. [20] as modified by Martinez-Saez et al. [21]. The digests were centrifuged at 10,000 rpm, at 4 °C for 20 min. The undigestible fractions obtained at this stage were kept at −80 °C for further analysis. Supernatants were cleaned by taking out bile salts. Finally, a soluble fraction was obtained which corresponded to the intestinal digests of BSG (DBSG) and EBSG (DEBSG), composing the duodenal bioaccessible nutrients and bioactive compounds. Experiments were performed in triplicate. The effect of pH changes was evaluated employing samples without enzyme addition.

#### 2.3.2. Glucose

The DBSG and DEBSG free glucose content was estimated by glucose oxidase/peroxidase reaction using a commercial kit (Spinreact, Girona, Spain). In a microplate, 5 µL of sample was mixed with 300 µL of glucose oxidase/peroxidase reagent. The mixture was incubated at 37 °C for 10 min. Finally, absorbance was measured at 505 nm using an Epoch 2 Microplate spectrophotometer (BioTek Winooski, VT, USA). A glucose calibration curve was used for quantification (0–11 mM). Analyses were performed in triplicate and the results expressed in mM.

#### 2.3.3. Amino Acids and Protein

##### Soluble Protein Content

The DBSG and DEBSG soluble protein content was determined using a bicinchoninic acid (BCA) microplate Pierce assay kit compatible with reducing agents based on Smith et al. [22]. Nine microliters of sample or standard were added to the center of the microplate well with 4 µL of compatible reagent solution. The plate was shaken for 1 min and was incubated for 15 min at 37 °C. Afterwards, 260 µL of BCA working reagent solution was added to each well and the microplate was incubated for 30 min at 37 °C. Finally, the plate was cooled at room temperature and absorbance was measured at 562 nm. A BSA calibration curve was undertaken for quantification (0–2 mg BSA/mL). Experiments were performed in triplicate. The protein digestibility percentage was calculated as follows [23]:

(1) $$Protein\;digestibility\;\left(\%\right)=\frac{Soluble\;protein\;\left(\frac{g}{100\;g\;of\;digested\;sample}\right)}{Protein\;content\;\left(\frac{g}{100\;g\;of\;sample}\right)}\times 100$$

##### Gluten Content

Gluten was determined for DBSG and DEBSG according to the enzyme-linked competitive immunoassay R5 method as described in Mena et al. [24].

##### Free Amino Acid Content

The total amino acids released during BSG and EBSG in vitro oral-gastro-intestinal digestion was determined by o-phthalaldehyde (OPA) assay as described by Shene et al. [25], adapted to a micro-method. OPA reagent was prepared by dissolving 5 mg of OPA in 100 µL of ethanol (96%), 5 µL of β-2- mercaptoethanol and 10 mL of 50 mM carbonate buffer (pH 10.5). In a quartz microplate, 50 µL of sample were mixed with 200 µL of OPA reagent. Absorbance was measured during 1 min at 340 nm using a microplate reader (BioTek Epoch 2 Microplate spectrophotometer, Winooski, VT, USA). Analyses were performed in triplicate. An *N*-acetyl-lysine (0–1.20 mM) curve was done for quantification and the results were expressed in mM of *N*-acetyl-lysine equivalents.

The amino acid (AA) profile of DBSG and DEBSG was determined. Half µL samples of DBSG and DEBSG were analyzed using a Biochrom30 series amino acid analyzer (Biochrom Ltd., Cambridge Science Park, Cambridge, UK), based on Spackman et al. [26]. The determinations were performed in triplicate and the results were expressed in mM of AA.

##### Advanced Glycation End Products (AGEs) Content

Fluorescent AGEs were measured as described by Martinez-Saez et al. [27]. One hundred and fifty µL of DBSG and DEBSG were added on a black microplate and fluorescence was measured using a microplate reader (BioTek Cytation5 Cell Imaging Multi-Mode Reader, Winooski, VT, USA) at 360 ± 40 nm and 460 ± 40 nm as the excitation and emission wavelengths, respectively. Analyses were performed in triplicate. A calibration curve was used for quantification with dihydrated quinine sulphate (0–640 µM).

#### 2.3.4. Phenolic Compounds

##### 2.3.4.1. Total Polyphenolic Content

Total phenolic content (TPC) was determined for duodenal digests (DBSG and DEBSG) by the Folin–Ciocalteu method based on Singleton et al. [28] adapted to micromethod [29]. A ferulic acid (FA) calibration curve was used for quantification (0–3.6 mM). Experiments were performed in triplicate and the results expressed as mM FA equivalent (FAeq) and mg of FAeq per gram of digested sample.

##### 2.3.4.2. Analysis of Phenolic Compounds by HPLC-QTOF Assay

DBSG and DEBSG phenolic compound identification by HPLC-QTOF was performed using HPLC equipment (Agilent 1200, Waldronn, Germany) equipped with a quaternary pump (G1311A), coupled degasser (G1322A), thermostated automatic injector (G1367B), thermostated column module (G1316A) and diode array detector (G1315B). The equipment was coupled to a mass spectrometer (Agilent G6530A Accurate Mass QTOF LC/MS) with an atmospheric pressure electrospray ionization source with JetStream technology. Control software used were the Masshunter Data Acquisition (B.05.00) and Masshunter Qualitative Analysis (B.07.00) programs. Samples and standard solution were injected (20 µL) in a ZORBAX Eclipse XDB-C18 column (150 mm × 4.6 mm × 5 µm) at 40 °C. The solvent systems were 0.1% formic acid (solvent A) and 0.1% formic acid diluted in acetonitrile (solvent B). The elution gradient (time, % of solvent A) was: 0 min, 95%; 20 min, 85%; 30 min, 70%; 35 min, 50%, 37 min, 95%, 45 min, 90%. Identification was performed by comparison of molecular formulae, retention times and previous references. A calibration curve for ferulic acid (1–16 µg/mL) was done. Semi quantitative results were obtained by comparing chromatogram areas for each compound and sample.

### 2.4. Colonic Bioaccessibility of Nutrients and Non-Nutrients

#### 2.4.1. In Vitro Simulation of Colonic Fermentation

Undigestible fractions obtained by applying the procedure in Section 2.3.1 were submitted to in vitro simulation of colonic fermentation based on several studies [30,31,32]. Fecal material was obtained from seven healthy volunteers who had not been treated with antibiotics for the last three months. Volunteers were provided with sterile containers, BD GasPak™ (NJ, USA) EZ Anaerobe Container System Sachets and two zip bags. Fecal samples were collected a minimum of 16 h prior to analysis and kept at 4 °C under anaerobic conditions until analysis. Experimentation took place in an anaerobic cabin (BACTRON Anaerobic Environmental Chamber, SHELLAB, OR, USA). The fecal inoculum was composed of equal parts of fecal material from each volunteer and phosphate buffer (0.1 M, pH 7) at 30% (*w/v*). It was homogenized on a Stomacher homogenizer (Stomacher 400 Circulator, SEWARD, Worthing, UK) for 10 min. Fermentation medium (pH 7) was made of peptone (15 g/L), cysteine (0.312 mg/L) and sodium sulfide (0.312 mg/L). Five hundred milligrams of undigested fractions were mixed with 7.5 mL of fermentation medium and 2 mL of fecal inoculum in a screw tap tube. The tubes were left under constant agitation for 24 h at 37 °C. Microbial activity was stopped by immersion in ice. Tubes were centrifuged at 4500 rpm for 10 min and supernatants were filtered (0.2 µm). The filtrate corresponded to the colonic fermented digests of BSG (FBSG) and EBSG (FEBSG). Aliquots were stored at −20 °C for further analysis. Experiments were performed in triplicate.

##### Microbiota Analysis

Microorganisms present in the fecal inoculum were determined immediately after collection, following Tamargo et al. [33]. Samples were diluted to different concentrations in physiological solution (0.9%). These dilutions were plated on different types of media: trypticase soy agar (TSA) (Difco™ BD, NJ, USA) for total aerobes; Wilkins Chalgren agar (Difco™ BD) for total anaerobes; MacConkey agar (Difco™ BD) for *Enterobacteriaceae*; Enterococcus agar (Difco™ BD) for *Enterococcus* spp.; MRS agar (pH = 5,4) (Pronadisa, CONDA, Madrid, Spain) for lactic acid bacteria; tryptose sulfite cycloserine agar (TSC) (Pronadisa, CONDA) for *Clostridium* spp.; BBL CHROMAgar (Difco™,BD) for *Staphylococcus* spp.; Bifidobacterium agar modified by Beerens (Difco™ BD) for *Bifidobacterium* spp. and LAMVAB for specific fecal *Lactobacillus* spp. [34]. All plates were incubated at 37 °C for 24 h to 72 h with an anaerobic gas pack system (BD, NJ, USA), except for BBL CHROMAgar and TSA, which were incubated in aerobic conditions. Plate culture was performed in triplicate and the results were expressed as the logarithm of colony forming units per mL [log(CFU)/mL].

#### 2.4.2. Sugars

Identification and quantification of sugars (glucose, fructose, arabinose, xylose) in FBSG and FEBSG was performed by ion exchange liquid chromatography (LC-IC). The equipment used consisted of a Metrohm Advanced Compact ion chromatographic instrument with Bioscan module (817 IC. Metrohm, Herisau, Switzerland), equipped with a pulse amperometric detector (PAD) (945 Professional Detector Vario, Metrohm), a pump (IC Pump 812), 889 IC Sample Center injector (Metrohm), and coupled with a degasser (IC-837, Metrohm). Between two and 100 µL of samples and D(+) glucose (1.08337.0250, Merck), D(−) fructose (1.04007.0250, Merck) and D(−) arabinose standards (375,763.1206, Sigma-Aldrich) were injected in a Metrosep Carb 2 column (250 × 4 mm). The mobile phase used was 300 mM sodium hydroxide and 1 mM sodium acetate, applied at a flow rate of 0.5 mL/min for 35 min. Identification was performed by comparison of the retention time with the patterns used. Data were analyzed using Metrodata IC Net 2.3 and MagiIC Net 2.3 (Metrohm, Herisau, Switzerland) software. Analyses were performed in triplicate.

#### 2.4.3. Organic Acids

A Metrohm Advanced Compact ion chromatography instrument (867 IC. Metrohm, Herisau, Switzerland) equipped with a conductivity detector (IC-819), 889 IC sample center injector, coupled with a degasser (IC-837, Metrohm, Herisau, Switzerland), was used for analysis of organic acids. FBSG, FEBSG, lactate standard (07096-100 ML, Sigma-Aldrich) and succinate standard (43057-100 ML, Sigma-Aldrich) were injected at a volume of 20 µL in a Metrosep organic acids column (250 × 4 mm, 5 µm). The mobile phase used was 0.5 mM sulfuric acid and 15% acetone, at 0.5 mL/min for 20 min. Identification was performed by comparison of the retention time with patterns used. The control software used were Metrodata IC Net 2.3 and MagiIC Net 2.3 (Metrohm, Herisau, Switzerland). Analyses were performed in triplicate.

#### 2.4.4. Short-Chain Fatty Acids

Short-chain fatty acids (SCFAs) were determined in colonic-fermented digests of BSG (FBSG) and EBSG (FEBSG). Fecal inoculum SCFAs were also determined to establish the baseline. Four hundred µL of samples or standards were acidified with 200 µL of phosphoric acid (0.5%). One hundred µL of methyl valeric (8092 µM) was added as an internal standard and was extracted with 1000 µL of n-butanol. Identification and quantification of SCFAs was performed using a gas chromatograph (Agilent 6890A, Santa Clara, CA, USA) equipped with a flame ionization detector (260 °C), an automatic injector (G2613A, Santa Clara, CA, USA) and a DB-WAXtr column (60 m × 0.325 mm × 0.25 μm) (Agilent Technologies, Santa Clara, CA, USA). Two µL of samples or standards were injected using a splitless method at 250 °C. The carrier gas used was helium at a constant flow rate (1.5 mL/min). The initial column temperature was 50 °C held for 2 min, increased to 150 °C at a rate of 15 °C/min, to 200 °C at 5 °C/min and then increased to 240 °C at a rate of 15 °C/min and kept for 20 min. The control software used was MSD Chemstation E.02.00.493 Agilent (Santa Clara, CA, USA). Analyses were performed in triplicate.

#### 2.4.5. Phenolic Compounds

Phenolic compounds of fermented colonic digests (FBSG and FEBSG) were analyzed by the Folin–Ciocalteu method as described in Section 2.3.4.1. The phenolic compounds were identified as reported in Section 2.3.4.2.

### 2.5. Bioactivity of Bioaccessible Compounds

#### 2.5.1. Antioxidant Capacity

##### ABTS Method

The BSG and EBSG intestinal and colonic fermented digests’ antioxidant capacity was measured using the ABTS method proposed by Re et al. [35] following the procedure described by Martínez-Saez et al. [21]. A ferulic acid calibration curve was used for quantification (0–80 µM). Experiments were performed in triplicate and the results expressed in mM FAeq and µmol FAeq/g of the digested sample

##### ORAC Method

Assessment of total antioxidant capacity by oxygen radical absorbance capacity (ORAC) was performed according to Ou et al. [36] for the BSG and EBSG intestinal- and colonic-fermented digests. A ferulic acid calibration curve was used for quantification (0–30 µM). All measurements were performed in triplicate and the results expressed as mM FAeq.

##### Intracellular Reactive Oxygen Species (ROS) Formation

Physiological intracellular reactive oxygen species (ROS) were measured on normal rat small intestine epithelial cells (IEC-6 cells) using a fluorescent probe DCFH-DA, as described by Iriondo-DeHond et al. [36], with slight modifications. Briefly, IEC-6 cells were seeded at a density of 2 × 10^4^ cells/well on a 96-well plate and cultured in complete medium (DMEM with 4.5 g/L of glucose, 10% *v/v* of FBS, 1% *v/v* of L-glutamine and 1% *v/v* of antibiotics) for 24 h (37 °C, 5% CO_2_). Afterwards, the medium was aspirated, and cells were loaded with 100 µL solution containing DBSG and DEBSG at a concentration of 15% *v/v* and medium without FBS, at a ratio equal to 1:10. Digests concentration (15% *v/v*) was not cytotoxic, as shown in (Supplementary Material Figure S1), determined by MTT assay [37]. Following a 24 h incubation period, the well contents were aspirated, and cells were pre-loaded with 100 µL of medium without FBS containing 2 µL of DCFH-DA (0.3 mg/mL in DMSO). Cells were incubated for 45 min. Afterwards, culture medium was removed, cells were washed with PBS and were treated with 100 µL containing DBSG (15% *v/v*) and DEBSG (15% *v/v*) and medium without FBS at a ratio equal to 1:10 for 30 min. Tert-butylhydroperoxide (tBOOH) 1 mM was used as an oxidation control and ferulic acid (10–100 µM) was used as an antioxidant control. Ferulic acid concentrations were shown to be non-cytotoxic; the results are shown in (Supplementary Material Figure S2). A control was set up by adding only medium without FBS to establish ROS formation under assay conditions. Afterwards, fluorescence was measured at 485 nm/528 nm (BioTek Synergy HT Multi-Mode Microplate Reader). Finally, 20 µL of MTT reagent were added per well and the plates were incubated for 90 min. Supernatants were removed, 100 µL of DMSO were added to each well and the absorbance measured at 570 nm using a BioTek Epoch 2 Microplate spectrophotometer (Winooski, VT, USA) [37]. All measurements were performed in triplicate and for three different cell passages. Intracellular ROS formation was calculated as follows:

(2) % ROS formation=FluorescenceSampleMTT AbsorbanceSample FluorescenceControlMTT AbsorbanceControl ×100 

#### 2.5.2. Anti-Inflammatory Properties

The anti-inflammatory properties of DBSG and DEBSG were determined by quantifying the nitrogen oxide (NO) production in macrophages (RAW264.7) as described by Benayad et al. [38]. Briefly, RAW264.7 cells were seeded on a 96-well plate (8 × 10^4^ cell/well) and cultured in complete medium (DMEM with 4.5 g/L of glucose, 10% *v/v* of FBS, 1% *v/v* of L-glutamine and 1% *v/v* of antibiotics) for 24 h (37 °C, 5% CO_2_). Afterwards, cells were treated with 150 µL of medium without FBS containing 1µg/mL lipopolysaccharide (LPS) from *Escherichia coli* O55:B5 and DBSG (15% *v/v*), DEBSG (15% *v/v*) or ferulic acid (10–100 µM). The intestinal digests and ferulic acid concentrations were shown not to be cytotoxic (Supplementary Material, Figure S3 and S4). Then, cells were incubated for 24 h (37 °C, 5% CO_2_). Negative and positive controls were tested consisting of medium without FBS and 1µg/mL of LPS in medium without FBS, respectively. After the incubation period, 100 µL of supernatants from the wells were removed and combined with 100 µL of Griess reagent (1% (*w/v*) sulfanilamide and 0.1% *w/v* N-1-(naphthyl)ethylenediamine-dihydrochloride in 2.5% *v/v* H_3_PO_4_). The mixtures were incubated at room temperature in the dark for 15 min and absorbance was measured at 550 nm in a BioTek Epoch 2 Microplate spectrophotometer (Winooski, VT, USA). A NO-in-DMEM-without FBS calibration curve was used for quantification (0–10 µg/mL). Analyses were performed in triplicate and for three different cell passages.

#### 2.5.3. Antidiabetic Properties

##### 2.5.3.1. Carbohydrase Activity

An enzymatic extract from rat intestine powder was obtained prior to analysis as described by Martínez-Saez [21]. The α-amylase, α-glucosidase and sucrase activity of rat intestinal extract was (424.02 ± 42.52) µmol of maltose × mL^−1^ × min^−^^1^, (0.181 ± 0.005) µmol of p-nitrophenyl × mL^−1^ × min^−1^ and (0.254 ± 0.028) µmol of glucose × mL^−1^ × min^−1^, respectively. The methods used are available in (Supplementary Material Figures S5–S7).

Assessment of DBSG and DEBSG’s α-glucosidase inhibitory activity was performed according to Lordan et al. [39], with slight modifications. Fifty µL of tested sample was mixed with 50 µL of 4-Nitrophenyl α-D-glucopyranoside (5 mM) and incubated at 37 °C for 5 min. An amount of 100 µL of rat intestinal extract (0.06 U/mL) was added to the previous mixtures. After incubating for 30 min at 37 °C, 80 µL of Na_2_CO_3_ 1 M was added. Absorbance was measured at 405 nm using a BioTek Epoch 2 Microplate spectrophotometer (Winooski, VT, USA). An acarbose calibration curve was done for quantification (0.00–0.30 mM).

Assessment of the intestinal digest α-amylase inhibitory activity was performed following Lordan et al. [39]. Briefly, 100 µL of tested sample or acarbose (0.001–0.25 mM) was incubated (10 min, 25 °C) with 100 µL of 1% potato starch. Afterwards, 100 µL of rat intestinal extract (40 U/mL) was added and the mixture was incubated (10 min, 25 °C) using an Eppendorf ThermoMixer™ C (Eppendorf, Madrid, Spain). Two hundred µL of DNS reagent was added and the mixture was incubated for 10 min at 100 °C. After cooling, 50 µL of sample was introduced in a microplate well, followed by 200 µL of distilled water. Absorbance was measured at 540 nm with a BioTek Epoch 2 Microplate spectrophotometer (Winooski, VT, USA).

Finally, a sucrase inhibitory activity assay was performed based on Li et al. [40]. Forty µL of sucrose (480 mM) was mixed with 10 µL of rat intestinal extract (0.254 U/mL) and 50 µL of tested sample. The mixture was incubated in an Eppendorf ThermoMixer™ C for 60 min at 37 °C. Afterwards, the mixture was heated at 100 °C for 10 min to stop the reaction. The concentration of glucose was measured as described in Section 2.3.2. An acarbose calibration curve was done for quantification (0.00–0.20 mM).

For all the inhibitory capacity experiments, a positive control with the addition of PBS instead of tested sample or acarbose was set up. All experiments were performed in triplicate. The results were expressed as mM of acarbose equivalents or by inhibition capacity measured as:

(3) $$\%\;inhibition=\frac{A_{+}-\left(A_{sample}-A_{blank}\right)}{A_{+}}\;\;\;\;$$

where, $A_{+}$ is the absorbance value for the positive control, $A_{sample}$ is the absorbance of the reaction mixture and $A_{blank}$ is the absorbance of the reaction mixture with PBS instead of enzymatic extract.

##### 2.5.3.2. Glucose Absorption

DBSG and DEBSG glucose absorption in IEC-6 was determined. Tested samples were DBSG (15% *v/v*) and DEBSG (15% *v/v*) and a control containing an equivalent amount of glucose content. The concentration of the intestinal digest was determined by MTT assay [37] to guarantee at least 80% cell viability as shown in (Supplementary Material Figure S1).

Transwell plates with polycarbonate inserts (Transwell^®®^ inserts, 0.4 µm pore size, 1.1 cm^2^) were used to estimate glucose transport across IEC-6 cell monolayers. Cells were seeded on 12-well Transwell plates with 7.6 × 10^4^ cells/well. Cells were grown (37 °C, 5% CO_2_) for 10 days, to differentiate. Five hundred µL and 1500 µL of medium on the apical and basolateral sides of each well were added, respectively. The medium used was formed by DMEM with 4.5 g/L of glucose, 10% *v/v* of FBS, 1% *v/v* of L-glutamine and 1% *v/v* of antibiotics and was changed within 48 h. Transepithelial electrical resistance (TEER) was measured using a Millicell-ERS device (Millipore, Zug, Switzerland) to evaluate monolayer integrity.

On the assay day, cells were washed with PBS 10 mM and pH 7.4. Afterwards, PBS was aspirated, and cells incubated (37 °C, 5% CO_2_) for 30 min with PBS in the absence of glucose. Next, PBS was aspirated from both sides of the Transwell and replaced by 500 µL of tested sample prepared in PBS in the apical side and 1500 µL of PBS in the basolateral side. Plates were incubated (37 °C, 5% CO_2_). Two hundred µL was removed from the basolateral side at different times (10 min, 30 min, 45 min, 60 min, 75 min, 90 min and 120 min) and the volume in the basolateral side was replaced with PBS.

After finishing the assay, a Lucifer yellow test was performed to evaluate the cell monolayer permeability [41,42,43]. Wells were washed with PBS and 200 µL of Lucifer yellow reagent (50 µM) was added in the apical side. One and a half mL of PBS was added in the basolateral side and plates were incubated for 1 h at 37 °C. Finally, 200 µL aliquots from the basolateral side were removed and the fluorescence was measured (485 nm/528 nm). The permeability percentage was calculated as the coefficient between the sample fluorescence and Lucifer yellow reagent (50 µM) fluorescence. Permeability less than 5% was considered adequate.

Glucose absorption was determined by measuring glucose concentration (see Section 2.3.2) in the basolateral side at different times. Glucose contents at different times were plotted graphically and the incremental area under the curve (IAUC) was calculated.

##### 2.5.3.3. Glucose Transport Inhibition

The inhibition capacity of DBSG and DEBSG over glucose transporters present in the IEC-6 cells was tested in the presence and absence of sodium. Assays were based on Fernández et al. [44] with modifications.

The procedure was the same as that described in Section 2.5.3.2 with slight differences. Two buffer solutions: PBS and potassium phosphate buffer (PK), were used for making the assay in the presence or absence of sodium, respectively. PBS (10 mM, pH 7.4) was prepared by dissolving in 1 L of distilled water, 8 g of NaCl, 0.2 g of KCl, 1.44 g of Na_2_HPO_4_ and 0.24 g of KH_2_PO_4_. PK (10 mM, pH 7.4) was prepared by mixing 10.4 g of KCl, 0.53 g of KH_2_PO_4_ and K_2_HPO_4_. Tested samples added to the wells were glucose (25 mM), glucose 25 mM + DBSG (15% *v/v*), glucose 25 mM + DEBSG (15% *v/v*), glucose 25 mM + ferulic acid 0.5 mM and glucose 25 mM with reference inhibitors (0.1 mM phloretin, 0.3 mM phloridzin). Every solution was prepared on PBS or PK depending on whether the experiment was in the presence or absence of sodium.

Glucose absorption was determined by measuring glucose concentration (see Section 2.3.2) in the basolateral side at different times. The glucose contents at different times were plotted graphically and the incremental area under the curve (IAUC) was calculated. The inhibition percentage was calculated as follows:

(4) $$\%\;Glucose\;transport\;inhibition=\;\frac{IAUC_{Control}-IAUC_{Glucose\;25\;\mathrm{mM}+\;tested\;sample}}{IAUC_{Control}}\times 100$$

where, $IAUC_{Control}$ corresponds to the incremental area under the curve of glucose (glucose 25 mM) and $IAUC_{Glucose\;25\;\mathrm{mM}+tested\;sample}$ is equal to the incremental area under the curve of glucose (25 mM) + DBSG, DEBSG, ferulic acid or reference inhibitors (phloridzin and phloretin).

- Determination of the kinetic mechanism of inhibition

The V_max_ and K_m_ values for DEBSG and ferulic acid inhibition mechanisms were determined for glucose uptake and transport in IEC-6 based on Manzano et al. [45].

After seeding and growing cells as described in Section 2.5.3.2, cells were washed with PBS. Five hundred µL and 1500 µL of PBS were added to the apical and basolateral sides, respectively. Cells were incubated for 30 min (37 °C, 5% CO_2_). Afterwards, PBS was aspirated from both sides of the well and different treatments were applied to the cells: 0.5–40 mM glucose, 0.5–40 mM glucose + DEBSG (15% *v/v*) and 0.5–40 mM glucose + ferulic acid (0.5 mM). Five hundred µL of each treatment was added in the apical side and 1500 µL of PBS in the basolateral side. Plates were incubated for 30 min (37 °C, 5% CO_2_). Finally, sampling was carried out by removing 200 µL from the apical and basolateral sides.

Glucose content was measured in the basolateral and apical aliquots by the glucose oxidase-peroxidase method (Section 2.3.2). Glucose uptake was calculated as the difference between glucose concentration load and the remaining glucose concentration in the apical and basolateral sides. Lineweaver–Burk plots were constructed for glucose uptake and transport to estimate V_max_ and K_m_.

### 2.6. Statistical Data Analysis

All data are reported as mean ± standard deviation. T-student tests and one-way analysis of variance (ANOVA), applying Tukey’s test, were used to determine significant differences between samples (α ≤ 0.05). Analyses were performed using XLSTAT Version 2011 (Addinsoft 1995–2010, France). Pearson correlation coefficients were obtained with a significance level of 0.05.

## 3. Results and Discussion

### 3.1. Duodenal Bioaccessibility of Nutrients

Data on bioaccessible amino acids, glucose, gluten and soluble protein are presented in Table 1. Although in vitro static models are over-simplistic and do not reproduce all dynamic aspects of the gastrointestinal tract, they have been shown to be useful in predicting macronutrient digestion [46].

Regarding glucose bioaccessibility, no significant differences (*p* > 0.05) were observed in the BSG intestinal digest (DBSG) and extruded BSG intestinal digest (DEBSG) bioaccessible glucose levels (Table 1). The glucose released during human oral-gastro-intestinal digestion depends on the free glucose content of the food matrix and on the enzymatic digestion of nutritional polysaccharides. α-Amylase found in the saliva and duodenum catalyzes the α-1,4-glucan bonds forming starch, maltodextrins and malto-oligosaccharides, followed by hydrolytic reactions catalyzed by α-glucosidase which liberate glucose residues [47]. The values obtained were within the range considered normal (4.4–6.1 mM) in the blood of humans under fasting conditions [48].

Protein digestibility is considered the most important determinant of protein quality in adults [10]. Extrusion increased BSG protein digestibility as a higher content (*p* < 0.05) of soluble protein and free amino acids was found in DEBSG than in DBSG (Table 1 and Table 2). This might be caused by denaturation of proteins and inactivation of antinutritional factors, such as trypsin inhibitors, during extrusion [10,11]. The EBSG protein digestibility percentage (Equation (1)) was 48.3% while that for BSG was 40.3%. Soluble protein was determined using a bicinchoninic acid (BCA) method, in which tripeptides and larger peptides react [49]. Although, BCA is a method for protein quantification, the results may be influenced by soluble peptides present in the digests due to enzymatic activity. In fact, the method has previously been used for peptide quantification [50]. Furthermore, BSG and EBSG contain gluten [8]. However, gluten was not found in the intestinal digests (Table 1). This data suggests that gluten was completely digested and implies that the extrusion process did not affect the digestibility of this protein.

The amino acid profiles for the BSG and EBSG intestinal digests are shown in Table 2. Both food matrices contained serine and alanine as the major non-essential amino acids (NEAAs), while leucine, valine and phenylalanine were the most abundant essential amino acids (EAAs) present in its intestinal digests. However, aspartic acid, cysteine histidine and methionine showed the lowest bioaccessibility. Protein nutritional value is dependent on the quantity, digestibility, and availability of EAAs [10]. The intestinal EBSG digests presented higher (*p* < 0.05) EAA content than the BSG intestinal digests (Table 2), suggesting EBSG is of better nutritional quality than BSG. Intestinal EBSG digests presented higher content (*p* < 0.05) of leucine, isoleucine and threonine than intestinal BSG digests. The differential muscle protein synthetic response is largely dependent on the post-prandial availability of EAA, particularly leucine [51]. However, DEBSG presented lower bioaccessible lysine (*p* < 0.05) than DBSG (Table 2). This might be a consequence of advanced glycation end-products (AGEs) formed during the digestion process [27], which were 82.605 ± 1.927 and 83.408 ± 2.349 µM of quinine sulphate for DBSG and DEBSG, respectively. Although no significant difference (*p* > 0.05) was found in the AGEs levels, a significant (*p* < 0.05) negative Pearson correlation was found between lysine and AGEs content present in BSG and EBSG intestinal digests. The reaction of EAA and sugars in the gut may cause a significant reduction in their bioaccessibility and health disorders of different kinds. In addition, the AGEs formed might contribute to the body pool of AGEs and, therefore, to an overall state of chronic oxidative stress [27].

### 3.2. Duodenal Bioaccessibility of Non-Nutrients

Many factors influence the bioaccessibility of polyphenols, such as the food matrix and interactions with other components. [52]. Table 3 shows the bioaccessible total phenolic content in the duodenum (DBSG and DEBSG) and in the colon (FBSG and FEBSG). The bioaccessible compound concentration in the BSG and EBSG intestinal digests were 2.492 ± 0.107 and 2.787± 0.122 mM of FAeq (*p* < 0.05), respectively.

A higher (*p* < 0.05) duodenal bioaccessibility of phenolic compounds was found after the extrusion process for BSG, when measured by the Folin–Ciocalteu method. Extrusion might have released bound phenolics due to breaking of conjugated moieties [53]. A higher bioaccessibility of phenolic acids after extrusion has previously been reported for mango bagasse [54]. However, the bioaccessibility of phenolic compounds after extrusion of brown rice and oat was decreased, while it remained unchanged for wheat [55].

These results are consistent with the phenolic profile presented in Table 4, which shows an increase in the duodenal bioaccessibility of ferulic acid, dihydrocaffeic acid, and particularly of benzoic acid, in DEBSG compared to DBSG. Ferulic acid was quantified as 0.007 ± 0.003 mg/100 g of the digested sample in DBSG and 0.021 ± 0.004 mg/100 g of the digested sample in DEBSG (*p* < 0.05). However, similar phenolic profiles were found for DBSG and DEBSG, with 2-(3-hydroxyphenyl) propionic acid the most abundant compound. Polyphenols are highly sensitive to mild alkaline conditions in the small intestine and most dietary polyphenols are degraded or transformed into other compounds at this stage [52]. Thus, it is relevant to identify which phenolic compounds are bioaccessible after undergoing oral-gastro-intestinal digestion.

### 3.3. Colonic Bioaccessibility of Metabolites Formed by Microbial Fermentation of Nutrients: Short-Chain Fatty Acids (SCFAs)

The gut microbiota, consisting of numerous microbial species, is considered a vital organ that plays a pivotal role in the host’s health [19]. Most of the contributions made by the gut microbiota to human physiology are related to microbial metabolism [56]. The fermentation of exogenous or endogenous substrates is of major importance for the host’s health through the production of a wide variety of metabolites [57]. To establish if BSG and EBSG are fermented by gut microbiota after oral-gastro-intestinal digestion, an in vitro colonic fermentation was applied using human feces.

Fecal inoculum was obtained using a pool of fecal samples from seven European volunteers, six women and one man, with a normal body mass index and between 18 and 54 years of age. Table 5 shows the characterization of the viable microbial populations in the fecal inoculum. Similar microbial distributions were found in the culture-dependent characterization of fecal inoculum made by Tamargo et al. [58]. Nevertheless, an analysis considering genomic sequences could have been performed to better understand the microorganisms present in the fecal inoculum. The dominant gut microbial phylum are Firmicutes, Bacteroidetes, Actinobacteria, Proteobacteria, Fusobacteria, and Verrucomicrobia, with the first two phyla listed representing 90% of gut microbiota [14,59]. *Clostridium* spp. and *Bifidobacterium* appeared to be the predominant orders in the fecal inoculum (Table 5), belonging to the Firmicutes and Actinobacteria phyla, respectively. Firmicutes spore-forming bacteria typically account for 64–78% of Western human microbiota [60]. Although the Actinobacteria phylum is proportionally less abundant [59], it has been found that subjects adopting a Mediterranean diet had a higher abundance of *Bifidobacterium* [61]. A lower proportion of bacteria belonging to the Proteobacteria phylum was identified, corresponding to the *Enterobacteriaceae*. Moreover, lower counts of total aerobes were expected, as gut microorganisms live in an anaerobic environment. However, some *Enterobacteriaceae* are anaerobic facultative and may have grown under aerobic conditions.

Table 6 presents the short-chain fatty acid (SCFA) content after in vitro fecal fermentation of BSG and EBSG undigested material. SCFAs are essential gut microbiota metabolites, produced mainly from the fermentation of non-digestible carbohydrates that become available to the gut microbiota. SCFAs impact on host health at cellular, tissue and organ levels by mechanisms related to gut barrier function, glucose homeostasis, immunomodulation and obesity [61].

The degradation of undigested polysaccharides involves a variety of hydrolytic enzymes that are not produced by the host. This is essential for providing bacteria with carbon and energy from released sugars [57]. BSG dietary fiber is mainly formed of cellulose, hemicellulose and lignin [4], which are substrates for microbial degradation. Furthermore, resistant starch, whose presence has been previously reported in BSG [8], is a potential source of energy for gut microbes leading to the production of gas and SCFAs [62]. Amounts of 0.839 ± 0.107 µM of glucose and 0.742 ± 0.082 µM of glucose (*p* > 0.05) were found in BSG colonic-fermented digest (FBSG) and EBSG colonic-fermented digest (FEBSG), which might be associated with cellulose or resistant starch degradation. Moreover, arabinose and xylose monosaccharides present in BSG hemicellulose were quantified as arabinose by 0.802 ± 0.007 µM in FBSG and 0.650 ± 0.096 µM in FEBSG (*p* > 0.05), as both coelute in the chromatogram. In fact, the digestibility of cellulose and hemicellulose in a group of seven women was estimated at 70% and 72%, respectively, showing that there is extensive degradation of these polysaccharides in dietary plant cell wall material [63]. Cellulolytic strains isolated from human feces have been classified as *Ruminococcus* spp., *Clostridium* spp., *Eubacterium* spp. and *Bacteroides* spp. [63], some of which were identified in the fecal inoculum. Hemicellulose degrading activities were associated with *Bacteroides* species [63]. No fructose content was identified in FBSG and FEBSG.

Sugars fermentation results in acetate, propionate and butyrate as the main fermentation end-products [57,61]. These were the major SCFAs obtained for the fermentation of BSG and EBSG (Table 6). Acetate, propionate and butyrate are expected to exist in a molar ratio of 60:20:20, respectively [61], while our studies showed a ratio of 45:28:27 (1:0.62:0.60) in FBSG and 42:29:29 (1:0.69:0.69) in FEBSG. The relative proportion of each SCFA depends on the substrate, microbiota composition and gut transit time [61]. Significant differences (*p* < 0.05) for acetate content in FBSG and FEBSG may be related to the higher content of insoluble dietary fiber in unextruded BSG (48.57 ± 0.15% in dry weight basis) [8]. However, it has been found that extrusion increased the metabolism of dietary fibers from grains by gut microbiota in vitro using fecal microbiota and in vivo in rat feeding studies [64]. Furthermore, in vitro fermentation in a batch human fecal model showed ratios of acetic:propionic:butyric equal to 65:23:11, 1:0.4:0.1 and 1:0.3:0.3 for BSG arabinoxylans. The results confirmed that arabinose was consumed during fermentation of BSG-derived products [65,66]. The abundance of *Bacteroides* spp. is associated with the production of propionate and acetate, while butyrate is produced mainly by the Firmicutes phylum [61]. Moreover, several studies have reported the ability of selected strains of *Lactobacillus* and *Bifidobacterium* to ferment arabinoxylans, resulting in propionate production [65]. Different physiological effects have been associated with these SCFAs. Acetic acid can induce apoptosis of cancerous cells and prevent DNA oxidative damage caused by hydrogen peroxide in epithelial cells of the distal colon. Propionate has been shown to increase satiety and improve glucose homeostasis. The presence of butyrate has been related to anti-inflammatory and anticarcinogenic effects [14,63].

Different pathways are involved in the biosynthesis of these major SCFAs from dietary fiber fermentation once pyruvate is produced from sugars. Propionate can be synthesized by two pathways involving succinate and lactate as intermediates [56,57,61]. Although lactate or succinate presence was not identified in FBSG and FEBSG, these intermediates may have been further metabolized into major metabolites, such as propionate [57]. In addition, in previous studies, lactic acid production was noted during the first twelve fermentation hours, which was then depleted over time [65]. Propionate could also have been formed through the propanendiol pathway after microbial transformation of deoxy-sugars [61]. Although traces of rhamnose have been found in BSG [4], this is not the major type of monosaccharide present.

The digestibility of proteins by the host is more variable than that of carbohydrates which leads to different amino acid composition available to the gut microbiota [14,56]. Similar to fiber fermentation, protein fermentation produces SCFAs accompanied by branch-chained fatty acids (BCFAs), ammonia, amines, hydrogen sulfide, phenols and indoles [67]. BCFAs (isobutyric and isovaleric) were found in FBSG and FEBSG (Table 6) to a low extent. BCFAs are reliable markers of proteolytic fermentation as they are produced exclusively through the fermentation of branched-chain amino acids [67]. A significant amount of these amino acids, especially leucine, was previously identified in BSG and EBSG [8]. In fact, a significantly higher (*p* < 0.05) amount of isovaleric acid (formed by leucine fermentation) than isobutyric acid was found in FBSG. Little is known about the effect of BCFAs on the host’s health. Previous studies suggested their capacity to modulate glucose and lipid metabolism in the liver as SCFAs. However, protein catabolism in the gut has potential negative implications, as toxic compounds can be released [56]. Nevertheless, carbohydrate presence alters amino acid utilization by microbes, reducing the uptake of some amino acids, such as tyrosine, and increasing the uptake of others, such as valine [67].

### 3.4. Colonic Bioaccessibility of Non-Nutrients

The results for bioaccessible polyphenols in the colon are presented in Table 3. The molar concentrations of phenolic compounds bioaccessible in the colon were 1.903 ± 0.101 and 2.009 ± 0.059 mM FA eq. As well as phenolic duodenal bioaccessibility, extrusion favored (*p* < 0.05) the release of bioactive compounds in the colon after microbial fermentation. To the best of our knowledge, there are no previous studies evaluating the effect of extrusion on phenolic colon bioaccessibility. Nevertheless, the extrusion of barley and oat increased the total tract bioaccessibility of dietary polyphenols in pigs [68].

The gut microbiota biotransforms polyphenols into metabolites that may have a greater biological activity than their precursor structures [15]. Microbiota enzymes can perform hydrolysis, dehydroxylation, demethylation and decarboxylation [52]. In the phenolic profile (Table 4), only benzoic acid and 2-(3-hydroxyphenyl) propionic acid were detected in FBSG and FEBSG, which may have been a result of microbial metabolism. Hydroxyphenyl propionic acids are a metabolite of phenolic acid microbial fermentation and are later transformed into benzoic acid [52]. Nevertheless, new phenolic metabolites might also have been formed after microbial metabolism, depending on the structure of polyphenols, that might have not been identified in this analysis.

Comparing the bioaccessibility at different digestion stages (Table 5), it was 32% higher in the colon than in the duodenum stage for both BSG and EBSG. The bioaccessibility of polyphenols present in cereal grains was 68% in the colon and 28% in the small intestine [69]. Most phenolic acids in BSG are bound to arabinoxylans by ester bonds [70], so microbial esterases are required during digestion to improve their bioaccessibility [15].

### 3.5. Bioactivity of Bioaccessible Compounds

#### 3.5.1. Antioxidant Capacity

The antioxidant capacity of BSG and EBSG digests, after in vitro oral-gastro-intestinal digestion and colonic fermentation, is shown in Table 7. EBSG presented higher (*p* < 0.05) antioxidant activity measured by ABTS and ORAC after oral-gastro-intestinal digestion. This may have been a consequence of the higher (*p* < 0.05) content of phenolic compounds present in DEBSG than in DBSG (Table 3). A slight significant correlation was found for antioxidant capacity measured by ABTS and the total phenolic content of intestinal digests (*p* < 0.05, 0.72), while no correlation was found between their total phenolic content and antioxidant capacity as measured by ORAC. Furthermore, extruded apple pomace and sorghum have also shown higher antioxidant capacity after in vitro gastrointestinal digestion than the untreated material, measured by ORAC and ABTS, respectively [71,72]. Regarding the colonic-fermented digests, significant differences (*p* < 0.05) were observed for the antioxidant capacity of FBSG and FEBSG measured by ABTS, but not by ORAC. Additionally, colonic-fermented digests presented higher (*p* < 0.05) antioxidant capacity than intestinal digests.

**Figure 1:**
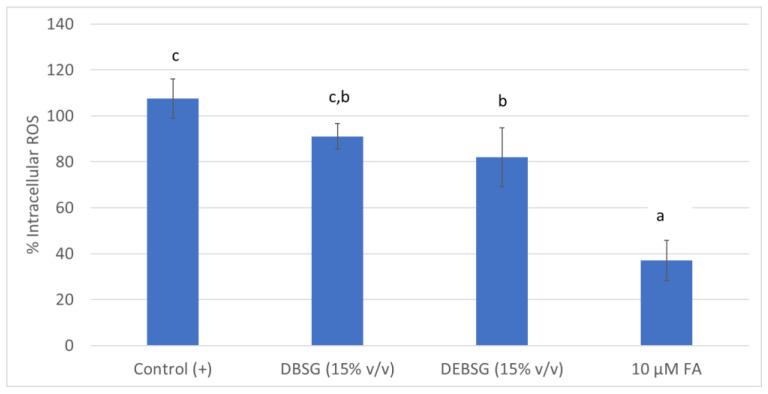
Intracellular ROS formation under physiological conditions in IEC-6 for untreated cells (Control (+)), cells treated with DBSG, cells treated with DEBSG and an antioxidant standard (FA). Percentage of ROS formation was calculated with respect to the Control (+). Different letters within columns show significant differences (*p* < 0.05). DBSG: brewers’ spent grain intestinal digest; DEBSG: extruded brewers’ spent grain intestinal digest; FA: ferulic acid.

Furthermore, intestinal digests (DBSG and DEBSG) were tested for their ability to inhibit intracellular ROS formation in IEC-6 (Figure 1). Cells treated with DEBSG showed a significant reduction (*p* < 0.05) in the formation of intracellular ROS under physiological conditions, i.e., in DEBSG-treated cells, ROS formation was 18% less than in untreated cells (control). This effect was not observed in cells treated with DBSG. This is in accordance with the higher amount of phenolic compounds (Table 3) with antioxidant capacity (Table 7) in DEBSG than DBSG. However, addition of 10 µM ferulic acid resulted in higher (*p* < 0.05) inhibition (70%) than for DBSG and DEBSG, although a dose-dependent effect was not observed for the tested concentrations (10–100 µM). Nevertheless, the results might also have been due to a higher content of soluble protein in DEBSG (Table 1). It has been found previously that BSG protein hydrolysates inhibited oxidative stress by reducing intracellular ROS formation in a Caco-2 cell line [73].

#### 3.5.2. Anti-Inflammatory Properties

**Figure 2:**
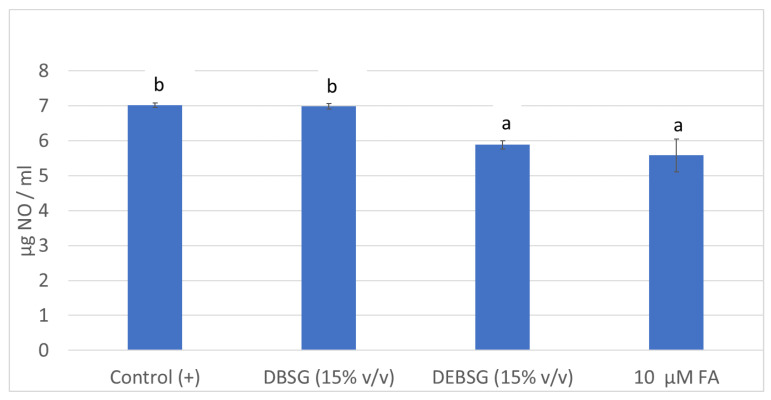
NO formation (µg/mL) in RAW 264.7 induced by LPS (1 µg/mL). Different letters within columns show significant differences (*p* < 0.05). DBSG: brewers’ spent grain intestinal digest; DEBSG: extruded brewers’ spent grain intestinal digest; FA: ferulic acid.

NO formation in RAW264.7 macrophages was determined as an inflammation biomarker after stimulation with LPS and coadministration with DBSG, DEBSG and ferulic acid (Figure 2). While DEBSG showed anti-inflammatory capacity under assay conditions, DBSG did not. In line with previous studies, greater anti-inflammatory properties have been recorded for extruded sorghum than unextruded sorghum on LPS-stimulated RAW264.7 [74]. Therefore, it may be inferred that the extrusion process favors the bioaccessibility of compounds with anti-inflammatory properties. This might be due to the greater (*p* < 0.05) release of phenolic compounds (Table 3) and soluble proteins (Table 1) in EBSG than in BSG after oral-gastro-intestinal digestion.

On the one hand, phenolic compounds are essential for the suppression of inflammation and their potent anti-inflammatory capacity has been shown [75]. A negative and significant correlation (*p* < 0.001, −0.94) was found between NO levels in cells treated with DBSG and DEBSG and their phenolic content measured by the Folin–Ciocalteu method. Furthermore, DEBSG exhibited statistically the same (*p* > 0.05) anti-inflammatory capacity than ferulic acid at the tested concentrations. Ferulic acid has already been reported to show anti-inflammatory effects inhibiting inducible nitric oxide synthase expression in LPS stimulated RAW264.7 cells, as well as NO production [76].

On the other hand, the anti-inflammatory effect of BSG protein hydrolysates has been previously described [77]. A negative significant correlation (*p* < 0.05, −0.73) was established between the soluble protein content of DBSG and DEBSG and NO levels. It was expected that, due to protein enzymatic hydrolysis during oral-gastro-intestinal digestion simulation, peptides might be solubilized into bioaccessible fractions which might be reflected in the BCA method results. Regarding mechanisms of action, studies have shown that protein hydrolysates limited proinflammatory cytokines and augmented anti-inflammatory IL-10 levels of spleen cells stimulated with LPS and Concanavalian A. Moreover, the involvement of both TLR2 and TLR4, and the main role of NFKB, in the immunomodulation by BSG hydrolysates has been demonstrated [78].

#### 3.5.3. Antidiabetic Properties

##### Carbohydrase Activity

Dietary starch hydrolysis, by α-amylase and α-glucosidase, is the major source of glucose in blood. Inhibition of these enzymes can significantly decrease the postprandial increase of blood glucose levels and therefore can be an important strategy in the management of hyperglycemia linked to type II diabetes [39].

Table 8 shows results obtained for DBSG and DEBSG α-amylase, α-glucosidase and sucrase activity of rat intestine acetone powder, as well as the acarbose concentration, which caused 50% inhibition of these enzymes’ activity (IC_50_). To our knowledge, this is the first investigation that has characterized α-amylase activity of rat acetone intestine powder. Previous studies suggested that a considerable amount of amylase seems to be formed in the rat small-intestinal mucosa itself [79]. The acarbose IC_50_ value for α-amylase activity was of the same order as that found in previous studies for human α-amylase (34.1 ± 0.8 µg/mL) [44] under similar experimental conditions. BSG and EBSG exhibited low α-amylase inhibitory activity which corresponded to 6.12% ± 0.31% and 12.44% ± 0.08%, respectively. These findings are consistent with previous studies which showed 13.35% ± 1.21% inhibition for porcine pancreatic α-amylase for BSG protein hydrolysates [80]. Additionally, different types of BSG acetone extracts showed porcine pancreatic α-amylase inhibitory capacity of 23.1% ± 4.2% and 49.7% ± 12.3% [47]. Regarding DBSG and DEBSG’s α-glucosidase inhibition capacity, it was significantly higher (*p* < 0.05) than that for α-amylase. BSG and EBSG intestinal digests showed strong α-glucosidase inhibition capacity equal to 57.71% ± 0.66% and 63.70% ± 1.04%, respectively. These results are in agreement with previous findings for BSG protein hydrolysates [80]. However, DBSG and DEBSG did not show sucrase inhibitory capacity under the assay experimental conditions.

The extrusion process of BSG showed a significant positive effect (*p* < 0.05) on its carbohydrase inhibition capacity. This might be due to the higher bioaccessibility (*p* < 0.05) of phenolic compounds (Table 3) and soluble protein (Table 1) present in EBSG. Previous findings suggested that ferulic acid, caffeic acid and isoferulic acid inhibited rat intestinal maltase activity in a mixed-inhibition manner, while no pancreatic α-amylase inhibition was found [81]. Furthermore, α-amylase and α-glucosidase inhibition effects of BSG protein hydrolysate have previously been reported [80,82]. Pearson correlation tests between α-glucosidase inhibition capacity and phenolic compounds or protein content in intestinal digests both resulted in a strong (Pearson coefficient ≥ 0.95) significant (*p* < 0.05) positive correlation. On the other hand, only intestinal digest protein content showed a significant (*p* < 0.05) correlation with α-amylase inhibition capacity, i.e., no correlation with phenolic content and α-amylase inhibition capacity of DBSG and DEBSG.

##### Glucose Absorption

**Figure 3:**
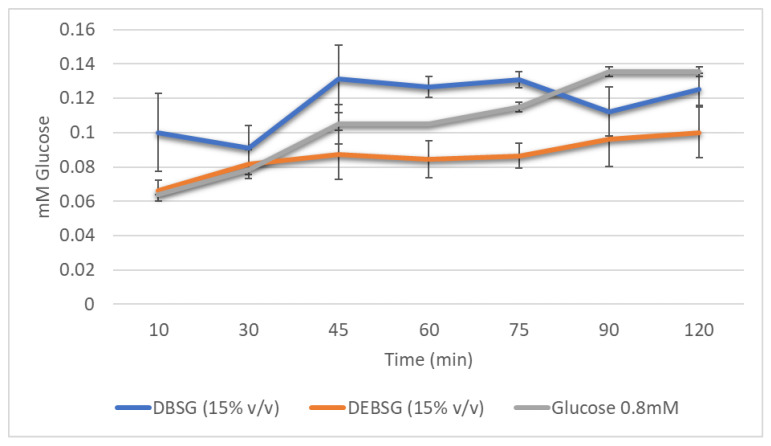
Brewers’ spent grain intestinal digests (DBSG) and extruded brewers’ spent grain intestinal digests (DEBSG) glucose absorption in IEC-6 cells over time.

Figure 3 shows glucose 0.8 mM DBSG and DEBSG glucose transport in IEC-6 over time. Glucose control absorption showed an acute peak at 45 min which decreased from 75 min onwards. Both, DBSG and DEBSG showed steadier glucose release into the basolateral side. Maximum glucose absorption for DBSG was registered after 90 min, while maximum glucose absorption for DEBSG was at 120 min. Therefore, use of BSG and EBSG may have implications for the prevention and management of chronic diseases such as type 2 diabetes. Carbohydrate-rich foods that promote sustained but low glucose levels, lower the glucose and insulin response and may have benefits for the metabolic control of diabetes and its complications [83]. Therefore, incorporating BSG as a food ingredient may contribute to satiety; glucose may play a role on the short-term satiety signal as maintained glucose plasma levels activate neuronal satiety pathways and contribute to satiety mechanisms by inhibiting gastric emptying [48].

Furthermore, the incremental areas under the curves (IAUC) presented in Figure 3 were 11.960 ± 0.058, 13.583 ± 0.386 and 10.129 ± 0.195 (mM × min) for glucose 0.8 mM, DBSG and DEBSG, respectively. There were significant differences (*p* < 0.05) between DEBSG’s IAUC and those for DBSG and glucose control, perhaps due to the higher bioaccessibility of phenolic compounds with glucose transporter inhibition capacity in DEBSG than in DBSG.

##### Glucose Absorption Inhibition

After polysaccharides are digested by pancreatic and intestinal enzymes, the resulting glucose cannot penetrate the lipidic intestinal cell membrane, so specific transporters are required. Two types of glucose transporter have been identified in human intestinal cells, as well as in rat enterocytes: sodium-dependent glucose transporters (SGLT1), which mediate glucose uptake into the cell at a 1:2 ratio, with sodium ions in favor of its electrochemical gradient, and a facilitated diffusion system (GLUT2). Classic theories have suggested that SGLT1 located in the brush border membrane mediates the uptake of glucose inside the cell, while GLUT2 transports it across the basolateral membrane to the blood. However, recent studies of the rat jejunum have suggested that, at high concentrations, luminal glucose (≥30 mM) saturates SGLT1 causing the translocation of GLUT2 into the apical membrane, increasing the capacity for glucose transport in the enterocyte [48,84,85,86,87,88,89].

Disturbed regulation of blood glucose levels can lead to hyperglycemia, which is a central problem in the pathophysiology of metabolic diseases, such as obesity, metabolic syndrome and type II diabetes [48]. Hence, controlling glucose levels after meals is a relevant strategy for the prevention and/or treatment of these chronic diseases [44].

**Figure 4:**
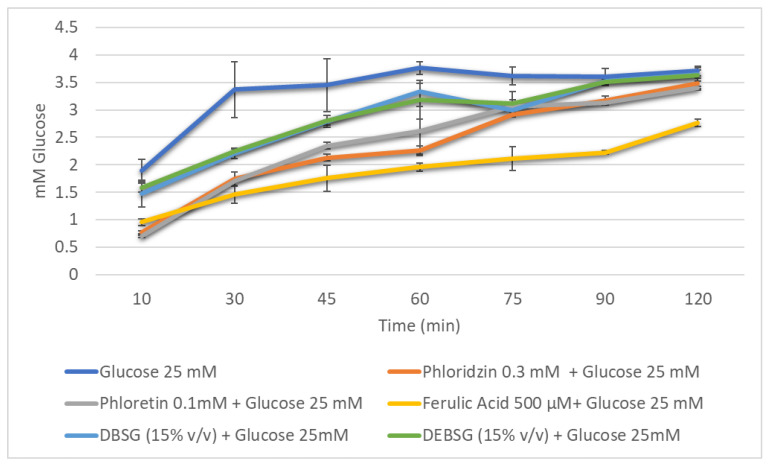
Glucose absorption in IEC-6 cells over time against reference inhibitors of glucose transporters (phloridzin and phloretin), ferulic acid, brewers’ spent grain intestinal digest (DBSG) and extruded brewers’ spent grain intestinal digest (DEBSG) under sodium-dependent conditions.

**Figure 5:**
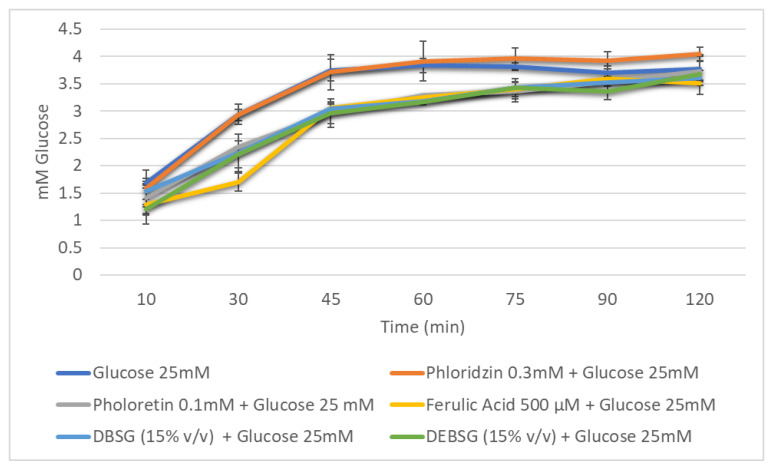
Glucose absorption in IEC-6 cells over time against reference inhibitors of glucose transporters (phloridzin and phloretin), ferulic acid, brewers’ spent grain intestinal digest (DBSG) and extruded brewers’ spent grain intestinal digest (DEBSG) under sodium-free conditions.

Glucose transport levels in IEC-6 over time in the presence of different inhibitors (phloridzin, phloretin), ferulic acid (500 µM), DBSG and DEBSG are presented in Figure 4 (sodium-dependent conditions) and Figure 5 (sodium-free conditions). Furthermore, the incremental areas under the curve (IAUCs) are presented in Table 9.

Under sodium-dependent conditions, it was expected that SGLT1 and GLUT2 transporters would be active, while under sodium-free conditions, only GLUT2 would be active. Phloridzin and phloretin, phloridzin aglycone, were used as standard inhibitors to check this. As calculated from Table 9, using Equation (4), phloretin inhibited glucose transport under sodium-dependent and sodium-free conditions by 27.3% and 12.1%, respectively. However, consistent with previous studies [90,91], no inhibition was observed for phloridzin under sodium-free conditions (Table 9), as this compound is supposed to inhibit SGLT1 transporter. Regarding ferulic acid, it presented a stronger inhibition of glucose transport under sodium-dependent conditions than under sodium-free conditions: 43.1% and 15.5%, respectively. Welsch et al. [90] reported a 38% ± 2.6% inhibition of glucose uptake under sodium-dependent conditions in rat intestinal brush border membrane vesicles, and Malunga et al. [92] suggested that ferulic acid inhibited glucose uptake in Caco-2 cells by interfering with GLUT2. Finally, to the best of our knowledge, this is the first time that brewers’ spent grain and extruded brewers’ spent grain intestinal digest glucose transport inhibition capacity has been studied. The results showed that DBSG and DEBSG exhibited the same glucose transport inhibition capacity as each other and under different experimental conditions (Table 9). Therefore, GLUT2 appears to have been the principal glucose transporter inhibited by DBSG and DEBSG. Antidiabetic properties have been associated with foods containing flavonoids and other phenolic compounds [88]. Li et al. [93] suggested that the mild glucose transport inhibition in Caco-2 cells caused by oat food digests may be due to a synergistic effect of all compounds present in the digests, including phenolic acids.

To better elucidate the inhibition mechanism, transporter kinetic parameters for IEC-6 glucose uptake and transport were determined for DEBSG and ferulic acid (Table 10). Regarding glucose uptake at the apical side, the glucose Km value was similar to that found by Zheng et al. [89] after 1 h of glucose starvation (18.5 ± 4.6 mM). Only ferulic acid showed a glucose uptake inhibition due to an increase of 61% in the Km and no statistical (*p* > 0.05) change in Vmax, owing to a competitive type of inhibition. However, both DEBSG and ferulic acid showed a non-competitive inhibition pattern for the transport of glucose in the basolateral side, the same type of inhibition as that reported for strawberry extracts [45]. Hence, different inhibition kinetics were found for DEBSG and ferulic acid. Further investigation is needed to establish which compounds present in DEBSG may be responsible for glucose transport inhibition.

## 4. Conclusions

In general, extruded BSG showed improved nutritional value and health-promoting properties compared to untreated BSG. The technological process did not affect glucose bioaccessibility or gluten digestibility (*p* > 0.05), while it favored amino acid release during digestion (*p* < 0.05). Acetate, propionate and butyrate, in a ratio of 45:28:27 and 42:29:29, were the major short-chain fatty acids generated by colonic fermentation of untreated BSG and extruded BSG dietary fiber, respectively. On the other hand, a higher (*p* < 0.05) gastrointestinal and colonic bioaccessibility of brewers’ spent grain phenolic compounds was achieved with the extrusion process. Extruded brewers’ spent grain intestinal digests inhibited glucose transport and showed higher (*p* < 0.05) α-amylase and α-glucosidase inhibition than untreated BSG. Moreover, lower (*p* < 0.05) glucose absorption was observed for extruded brewers’ spent grain than for untreated brewers’ spent grain. Furthermore, extruded brewers’ spent grain intestinal digests inhibited intracellular ROS formation and had anti-inflammatory properties, while untreated brewers’ spent grain did not. Therefore, extruded brewers’ spent grain appears to represent an interesting sustainable ingredient with potential biological properties that might help treating and preventing non-communicable diseases, especially those dependent on energetic metabolism.

## Figures and Tables

**Table 1:** Bioaccessibility of glucose, gluten, free amino acids and soluble protein in duodenal digests.

	Glucose (mM)	Soluble Protein (mg BSA/mL)	Gluten (ppm)	Amino Acids (mM of Equivalent *N*-acetyl lysine)
DBSG	4.654 ± 0.347 ^a^	17.089 ± 0.069 ^a^	<3 ^a^	55.781 ± 3.516 ^a^
DEBSG	4.788 ± 0.217 ^a^	18.942 ± 0.012 ^b^	<3 ^a^	69.776 ± 6.389 ^b^

Different letters within the same column show significant differences (*p* < 0.05). BSA: bovine serum albumin; AGEs: advanced glycation end products; DSG: brewers’ spent grain intestinal digest; DEBSG: extruded brewers’ spent grain intestinal digest.

**Table 2:** Amino acid profile for BSG intestinal digests (DBSG) and EBSG intestinal digests (DEBSG).

	Amino Acid (mM)	DBSG	DEBSG
Non-essential amino acids (NEAA)	Alanine (Ala)	5.759 ± 0.220 ^a,J^	6.719 ± 0.353 ^b,K^
	Arginine (Arg)	3.442 ± 0.038 ^a,G,H^	3.363 ± 0.232 ^a,I^
	Aspartic acid (Asp)	0.235 ± 0.025 ^a,A^	0.235 ± 0.025 ^a,A^
	Cysteine (Cys)	0.502 ± 0.034 ^a,A^	0.461 ± 0.075 ^a,A,B^
	Glutamic acid (Glu)	1.309 ± 0.070 ^a,C,D^	1.328 ± 0.098 ^a,C,D,E^
	Glycine (Gly)	3.259 ± 0.075 ^a,G^	3.294 ± 0.174 ^a,I^
	Proline (Pro)	1.535 ± 0.332 ^a,D^	1.578 ± 0.351 ^a,D,E,F^
	Serine (Ser)	8.762 ± 0.242 ^a,L^	10.498 ± 0.382 ^b,N^
	Tyrosine (Tyr)	1.600 ± 0.098 ^a,D^	1.788 ± 0.096 ^b,E,F^
Total NEAA		26.282 ± 0.717 ^a,^*	28.729 ± 1.788 ^b^
Essential amino acids (EAA)	Histidine (His)	1.139 ± 0.034 ^a,B,C^	1.158± 0.455 ^a,C,D^
	Isoleucine (Ile)	2.683 ± 0.092 ^a,F^	2.870 ± 0.133 ^b,H,I^
	Leucine (Leu)	7.360 ± 0.249 ^a,K^	8.729 ± 0.471 ^b,M^
	Lysine (Lys)	2.744 ± 0.060 ^b,F^	2.580 ± 0.154 ^a,H,G^
	Methionine (Met)	0.876 ± 0.021 ^a,B^	0.892 ± 0.068 ^a,B,C^
	Phenylalanine (Phe)	3.632 ± 0.189 ^a,H^	3.909 ± 0.295 ^a,J^
	Threonine (Thr)	1.975 ± 0.049 ^a,E^	2.079 ± 0.081 ^b,F,G^
	Tryptophan (Trp)	n.d.	n.d.
	Valine (Val)	4.541 ± 0.132 ^a,I^	4.824 ± 0.400 ^a,L^
Total EAA		24.947 ± 0.661 ^a^	27.041 ± 1.570 ^b^
Total		51.457 ± 1.230 ^a^	56.233 ± 3.074 ^b^

Different letters within the same line (lower case) and column (capital letters) show significant differences (*p* < 0.05). * shows significant differences between Total NEAA and EAA content for each sample. DSG: brewers’ spent grain intestinal digest; DEBSG: extruded brewers’ spent grain intestinal digests.

**Table 3:** Total polyphenolic content for BSG and EBSG intestinal- and colonic-fermented digests.

Total Phenolic Content (mg FAeq/g of Digested Sample)		
	BSG	EBSG
Duodenal bioaccessibility	3.221 ± 0.116 ^a,A^	3.604 ± 0.111 ^b,A^
Colon bioaccessibility	4.743 ± 0.235 ^a,B^	5.290 ± 0.072 ^b,B^

Different letters (lower case for rows and capital letter for columns) within the same column show significant differences (*p* < 0.05). BSG: brewers’ spent grain; EBSG: extruded brewers’ spent grain. FA: ferulic acid.

**Table 4:** Bioaccessibility of individual polyphenols in duodenal and colonic digests.

Proposed Compound	Molecular Formula	Molar Mass (g/mol)	Retention Time (min)	Duodenal Bioaccessibility			Colon Bioaccessibility		
				DBSG ^1^ (%)	DEBSG ^1^ (%)	Variation after Extrusion (%) ^2^	FBSG ^1^ (%)	FEBSG ^1^ (%)	Variation after Extrusion (%) ^2^
2-(3-hydroxyphenyl) propionic acid	C_9_H_10_O_3_	165.1	16.7	95.0	94.3	−3	96	96	14
Ferulic acid	C_15_H_18_O_8_	193.1	20.0	0.5	0.5	3	0	0	0
Dihydrocaffeic acid	C_9_H_10_O_4_	181.1	6.7	2.8	2.8	2	0	0	0
Benzoic acid	C_7_H_6_O_2_	121.0	11.5	1.8	2.4	31	4	4	0

(^1^) Percentage of chromatogram peak area respect total chromatogram area, (^2^) Percentage of variation due to extrusion process. DSG: brewers’ spent grain intestinal digest; DEBSG: extruded brewers’ spent grain intestinal digest; FBSG: brewers’ spent grain colonic fermented digest; FEBSG: extruded brewers spent grain colonic fermented digest.

**Table 5:** Microbial count in fecal inoculum.

	log (CFU/mL)	Relative Percentage to Total Anaerobes
Total anaerobes	9.575 ± 0.077 ^d^	100.00%
Total aerobes	7.504 ± 0.035 ^a,b^	0.842%
Enterobacteriaceae	7.435 ± 0.094 ^a,b^	0.728%
*Staphylococcus* spp.	7.155 ± 0.045 ^a^	0.377%
Lactic acid bacteria	8.354 ± 0.039 ^b,c^	5.965%
*Lactobacillus* spp.	7.110 ± 0.067 ^a^	0.342%
*Clostridium* spp.	9.357 ± 0.157 ^d^	62.281%
*Entereococcus* spp.	7.385 ± 0.027 ^a,b^	0.640%
Bifidobacteria	9.290 ± 0.061 ^d,c^	51.754%

Different letters within the same column show significant differences (Δlog > 1). CFU: colony forming units.

**Table 6:** Short-chain fatty acids and organic acids present in FBSG and FEBSG.

Short-Chain Fatty Acids (mM)		
	FBSG	FEBSG
Acetic acid	9.471 ± 0.139 ^b,D^	8.185 ± 0.674 ^a,C^
Propionic acid	5.808 ± 0.287 ^a,C^	5.739 ± 0.158 ^a,B^
Butyric acid	5.747 ± 0.142 ^a,C^	5.551 ± 0.216 ^a,B^
Isobutyric acid	0.302 ± 0.012 ^a,A^	0.284 ± 0.001 ^a,A^
Isovaleric acid	1.084 ± 0.048 ^a,B^	1.017 ± 0.090 ^a,A^
Valeric acid	1.008 ± 0.128 ^a,B^	0.837 ± 0.208 ^a,A^
Caproic acid	0.328 ± 0.036 ^a,A^	0.298 ± 0.085 ^a,A^
Total	23.748 ±0.181 ^b^	21.895 ± 1.035 ^a^

Different letters within the same line (lower case) and column (capital letters) show significant differences (*p* < 0.05). FSG: brewers’ spent grain colonic fermented digest; FEBSG: extruded brewers’ spent grain colonic-fermented digest.

**Table 7:** Antioxidant capacity for BSG and EBSG intestinal and colonic fermented digests.

	ABTS (mM FAeq)		ORAC (mM FAeq)	
	Duodenal	Colonic	Duodenal	Colonic
BSG	2.899 ± 0.158 ^a^	1.967 ± 0.081 ^a^	10.809 ± 0.109 ^a^	1.624 ± 0.044 ^a^
EBSG	3.232 ± 0.089 ^b^	2.132 ± 0.088 ^b^	13.000 ± 0.080 ^b^	1.785 ± 0.053 ^a^
	ABTS (µmol FAeq/g of digested sample)		ORAC (µmol FAeq/g of digested sample)	
	BSG	EBSG	BSG	EBSG
Duodenal	19.916 ± 1.170 ^A^	23.900 ± 0.673 ^A^	12.487 ± 0.344 ^A^	16.950 ± 0.195 ^A^
Colonic	25.354 ± 0.920 ^B^	28.184 ± 1.157 ^B^	21.262 ± 0.262 ^B^	21.632 ± 0.210 ^B^

Different letters (lower case for results in mM FA and capital letter for results in µmol FA/g) within the same column show significant differences (*p* < 0.05). BSG: brewers’ spent grain; EBSG: extruded brewers’ spent grain; FA: ferulic acid.

**Table 8:** Inhibition of digestive carbohydrases.

	IC50 Acarbose	DBSG	DEBSG
α-amylase (µM Acarbose)	10.522	0.435 ± 0.010 ^a,A^	0.689± 0.004 ^b,A^
α-glucosidase (mM Acarbose)	1.002	3.005 ± 0.123 ^a,B^	7.829 ± 1.560 ^b,B^
Sucrase (µM Acarbose)	15.152	n.i.d.	n.i.d.

Different letters within the same row (lower case) and column (capital letters) show significant differences (*p* < 0.05). n.i.d means no inhibition determined; DBSG: brewers’ spent grain intestinal digest; DEBSG: extruded brewers’ spent grain intestinal digest.

**Table 9:** Incremental area under the curve for glucose absorption inhibition models.

	IAUC Sodium Dependent Conditions (mM × min)	IAUC Sodium Free Conditions (mM × min)
Glucose 25 mM	377.153 ± 21.049 ^d,A^	380.010 ± 8.862 ^b,A^
Phloridzin 3 mM + Glucose 25 mM	272.741 ± 7.324 ^b,A^	381.344 ± 20.569 ^b,B^
Phloretin 1 mM + Glucose 25 mM	274.627 ± 6.602 ^b,A^	334.700 ± 7.394 ^a,B^
Ferulic acid 500µM + Glucose 25 mM	213.868 ± 11.630 ^a,A^	321.482 ± 7.037 ^a,B^
DBSG (15% *v/v*) + Glucose 25 mM	322.757 ± 1.854 ^c,A^	336.658 ± 9.084 ^a,A^
DEBSG (15% *v/v*) + Glucose 25 mM	324.793 ± 4.320 ^c,A^	324.747 ± 10.224 ^a,A^

Different letters within the same row (capital letters) or column (lower case) show significant differences (*p* < 0.05). DBSG: brewers’ spent grain intestinal digest; DEBSG: extruded brewers’ spent grain intestinal digest.

**Table 10:** Glucose uptake and transport kinetics in IEC-6 cells in presence of EBSG and ferulic acid.

	Km (mM)	Vmax (mM/min)	Inhibition
Uptake			
Control (Glucose)	19.490 ± 3.156	0.171 ± 0.027	n.a.
DEBSG (15% *v/v*)	16.273 ± 0.557	0.180 ± 0.029	No
Ferulic acid 500 µM	31.493 ± 3.646 *	0.336 ± 0.056	Competitive
Transport			
Control (Glucose)	128.427 ± 13.830	0.632 ± 0.003	n.a.
DEBSG (15% *v/v*)	97.726 ± 1.509	0.478 ± 0.015 *	Non-competitive
Ferulic acid 500 µM	133.578± 19.526	0.546 ± 0.021 *	Non-competitive

* Different letters within a column shows significant differences (*p* < 0.05) with the control value. n.a means not applicable; DEBSG: extruded brewers’ spent grain intestinal digest.

## Data Availability

The data presented in this study are available in the present article and in the supplementary material provided.

## Supplementary Materials

The following supporting information can be downloaded at: https://www.mdpi.com/article/10.3390/nu14173480/s1. Figure S1: IEC-6 viability for DBSG and DEBSG. *shows significant differences with positive control (C+) (*p* < 0.05). Figure S2: IEC-6 viability for ferulic acid. Figure S3: RAW 264.7 viability for DBSG and DEBSG. *shows significant differences with positive control (C+) (*p* < 0.05). Figure S4: RAW264.7 viability for ferulic acid. Figure S5: Maltose released from potato starch due to α-amylase activity of rat intestinal extract. Figure S6: p-Nitrophenyl released due to α-glucosidase activity of rat intestinal extract. Figure S7: Glucose released from sucrose due to sucrose activity of rat intestinal extract.
