# Supplementary material for: In Vitro Digestibility and Bioaccessibility of Nutrients and Non-Nutrients Composing Extruded Brewers’ Spent Grain
Source: Nutrients. 2022 Aug 24;14(17):3480. doi: 10.3390/nu14173480 (PMC9459946; doi:10.3390/nu14173480)
Supplement: Supplementary file 1 [file nutrients-14-03480-s001.zip › nutrients-1854578-supplementary.pdf]

Cell Culture

IEC-6 and RAW264.7 cells were cultured in Dulbecco´s modified Eagle's medium (DMEM) containing 1% penicillin-streptomycin, 1% L-glutamine and 10% heat-inactivated fetal bovine serum. Incubation conditions were 37°C, 5% CO_2 and 100% relative humidity.

Cell viability

Prior to studies involving cells (glucose transport, intracellular ROS formation and NO assay), cell's viability was tested by 3-(4,5-dimethylthiazol-2-yl)-2,5-diphenyltetrazole bromide (MTT) method to select the concentration of intestinal digests that guaranteed at least 80% viability in both cell lines used. MTT method was done according to (Bakondi et al., 2003). Cells were seeded on sterile 96-well plates in a density of 20000 cells/well and 80000 cells/well for IEC-6 and RAW264.7, respectively. Cells were incubated for 24 hours (37°C, 5% CO_2). Then, different concentrations of intestinal digests or ferulic acid were prepared in PBS, which were afterwards added to medium without FBS at a ratio equal to 1:10. Positive (PBS) and negative (DMSO 50%) controls were also analyzed. Treatments were performed by adding 100 µL of samples to the cells in absence of the culture medium for 24 hours (37°C, 5% CO2). Finally, 20 µL of MTT (6mM) reagent were added to each well and plates were incubated for 90 minutes. Afterwards, supernatants were removed and 100 µL of dimethyl-sulfoxide (DMSO) were added to each well and homogenized. Absorbance was measured at 570 nm in a microplate reader. Viability was calculated by considering the absorbance of the positive control (PBS treatment) as 100% viability. Experiments were performed in triplicate and for at least two different cell passages.

Results were expressed as mean ± standard deviation and are presented in Figure S1-S4. Figure S1 shows IEC-6 cells viability for different concentrations DBSG and DEBSG. Figure S2 shows the IEC-6 viability for different concentrations of ferulic acid. Figure S3 shows RAW264.7 cells viability for different concentrations of DBSG and DEBSG. Figure S4 shows RAW264.7 cell viability for different concentrations of ferulic acid. One way analysis of variance (ANOVA) was performed on each assay, and differences between samples were determined by the Tukey test ($\alpha \leq 0.05$).

These results allowed to choose no cytotoxic dose of samples and ferulic acid for treating IEC-6 and RAW2464.7 cells employed in the bioactivity assays. Dose causing at least 80% of cell viability compared to the positive control of viability (cells growing in physiological conditions without any treatment) were employed for evaluating their bioactive properties. DBSG and DEBSG intestinal digests were diluted 15% (v/v) to achieve this goal.

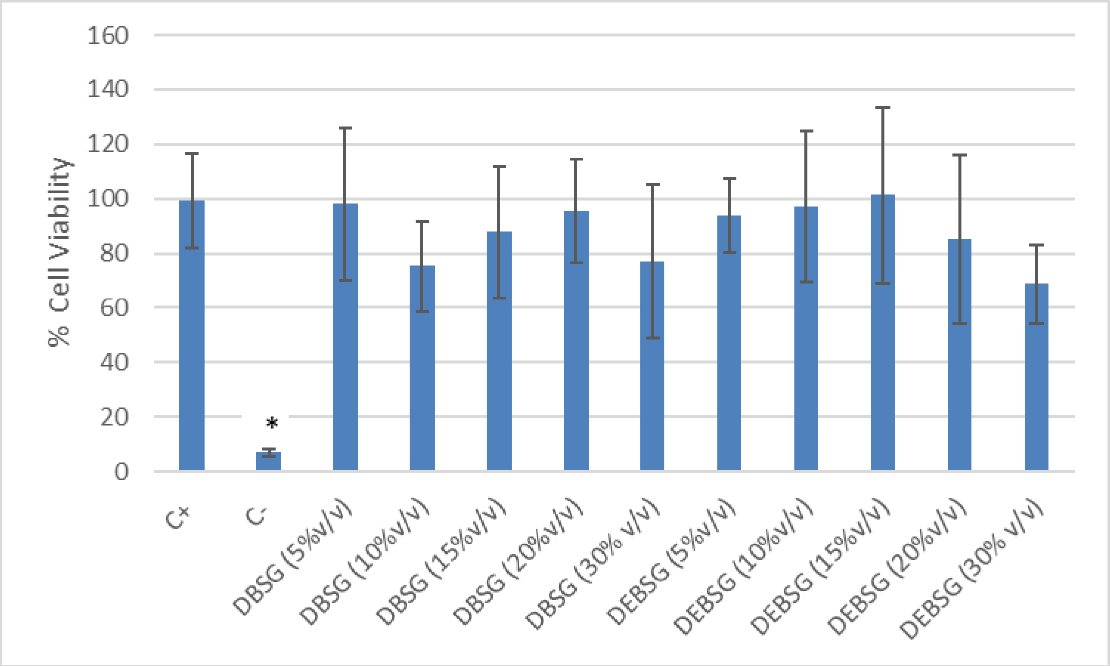

Figure S1- IEC-6 viability for DBSG and DBSGE. * shows significant differences with positive control (C+) ($p<0.05$).

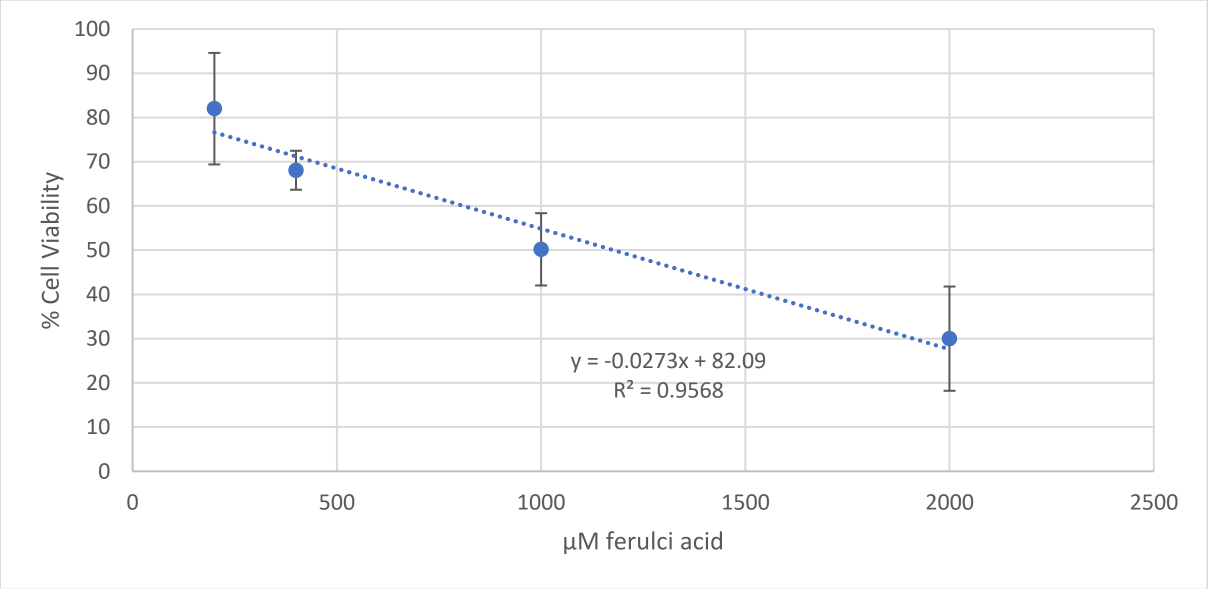

Figure S2- IEC-6 viability for ferulic acid

IC_{50} for ferulic acid was determined at 1175.45 µM (concentration in well).

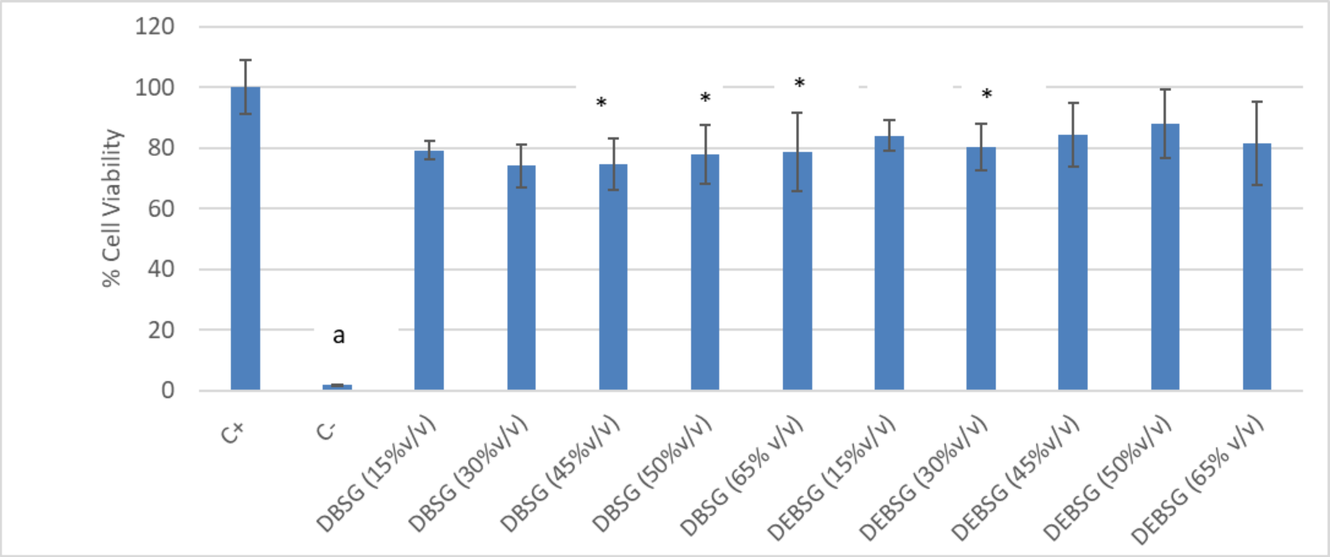

Figure S3- RAW 264.7 viability for DBSG and DBSGE. * shows significant differences with positive control (C+) ($p<0.05$), letter within column shows significant differences ($p < 0.05$).

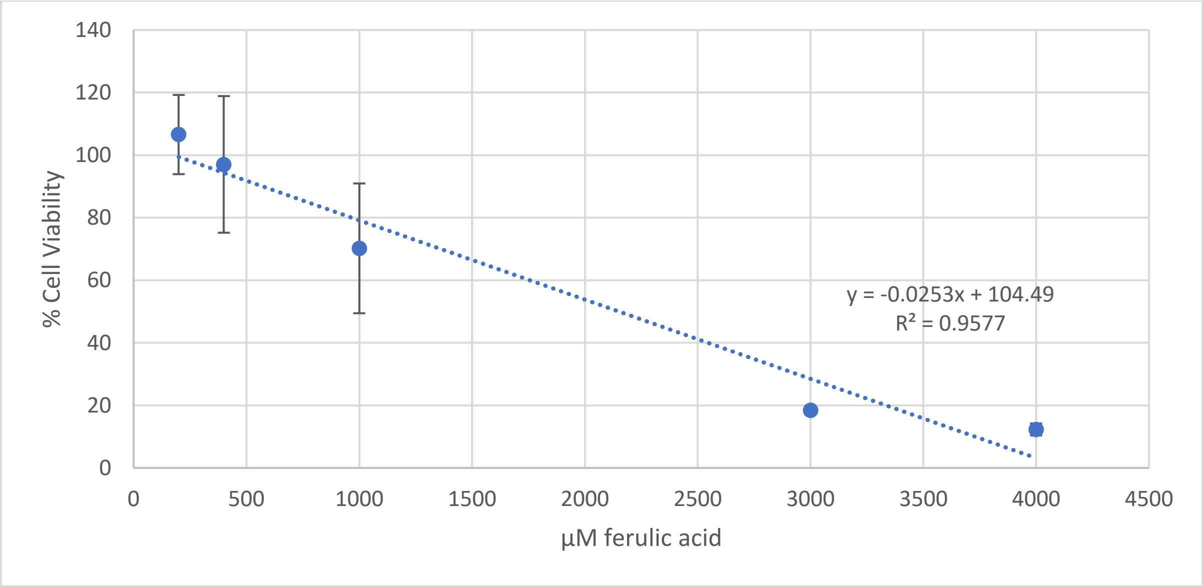

Figure S4- RAW264.7 viability for ferulic acid

IC_{50} for ferulic acid was determined at 2153.75 µM (concentration in well).

Carbohydrases' activity from rat intestinal acetone powder used for assessing the antidiabetic properties.

Characterization of α-amylase activity in rat intestine acetone powder

α-Amylase activity was determined based on enzymatic assay of α-Amylase (EC 3.2.1.1) from Sigma. One hundred µL of potato starch 1% (w/v) in saline buffer phosphate (20 mM, pH 7) were mixed with different volumes of diluted (1:10) rat acetone enzymatic extract ranging from 0 to 100 µL. Mixtures were incubated for3 minutes at 20°C. Afterwards 100 µL of DNS were added, followed by 15 minutes incubation at 100°C. Mixtures were cooled on ice-water bath and 900 µL of distilled water were added. Finally, enzymatic extract volumes were added to reach 100 µL of enzymatic extract. Absorbance was measured in a microplate (200 µL/well) at 540 nm. A maltose calibration curve was constructed for quantification (0.0 - 2.5 mg/ml). Figure S5 shows maltose released from potato starch by different volumes of rat intestinal extract. for the characterization of α-amylase activity from rat intestinal extract.

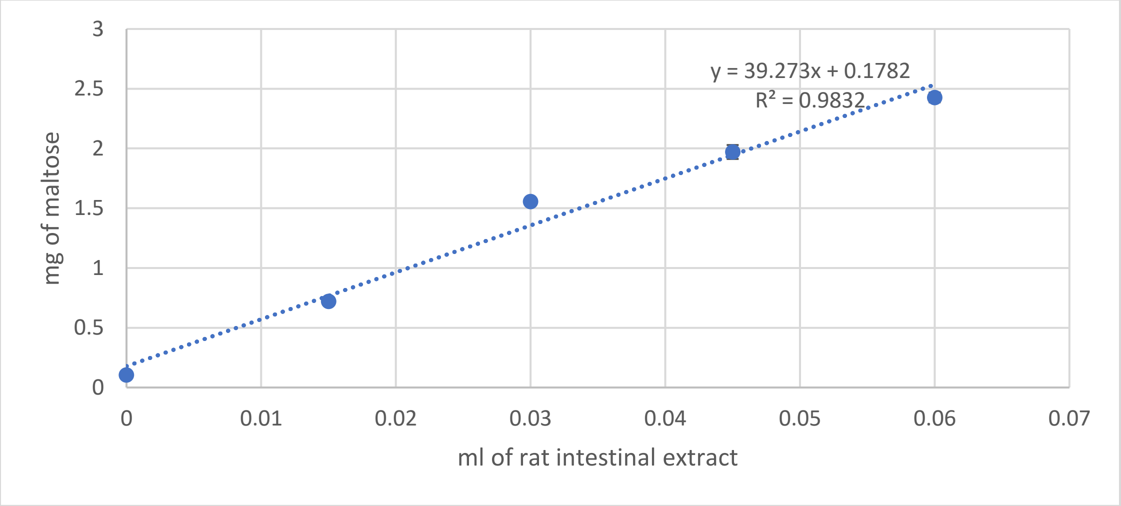

Figure S5- Maltose released from potato starch due to α-amylase activity of rat intestinal extract.

α-Amylase activity of the intestinal extract was of 424.02 ± 42.52 µmol of maltose×mL^{-1}×min^{-}

Characterization of α-glucosidase activity in rat intestine acetone powder

To assess the α-glucosidase activity in rat intestine acetone powder 50 µL of PBS (10 mM, pH 7) were mixed with 50 µL of 4-Nitrophenyl α-D-glucopyranoside (5 mM) and incubated for 5 minutes at 37°C. Then different enzyme concentrations were added, followed by a further incubation (30 minutes, 37°C). Finally, 80 µL of Na_2CO_3 (1M) were added to end up the reaction. Absorbance was measured at 405 nm. A p-nitrophenyl calibration curve was done (0 -1 mM).

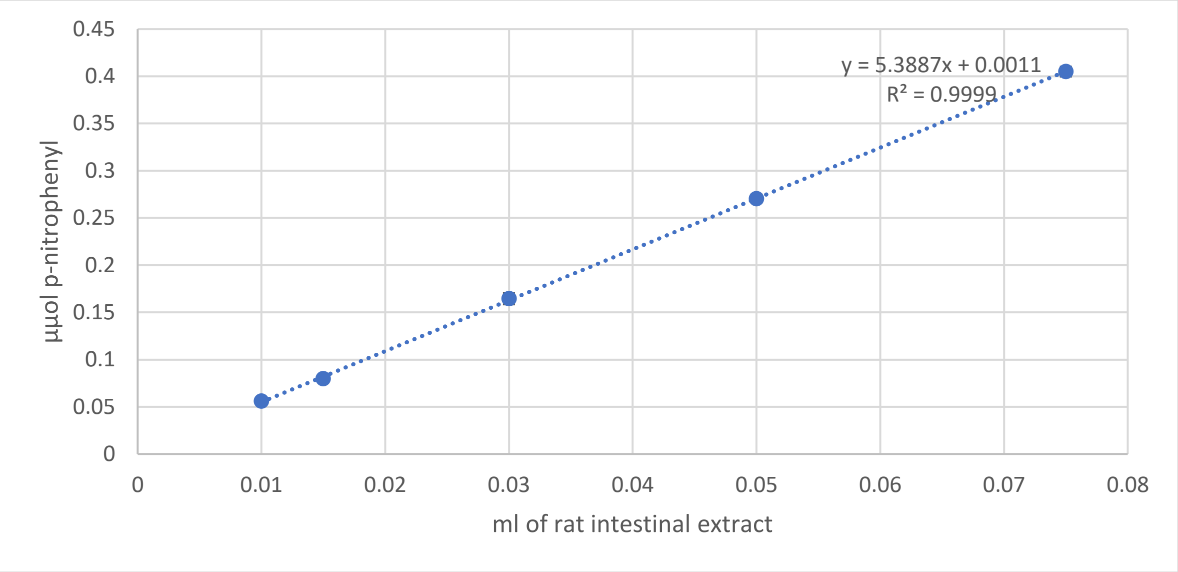

Figure S6- p-Nitrophenyl released due to α-glucosidase activity of rat intestinal extract.

α-Glucosidase activity was of 0.181 ± 0.005 µmol of p-nitrophenyl ×mL^{-1}×min^{-1}

Characterization of sucrase activity in rat intestine acetone powder

Sucrase activity of rat acetone intestine powder was determined as described by (Li, Chuang, & Hsieh, 2019), with slight modification. Different concentrations of enzymatic extract were prepared in PBS (10 mM, pH 7). Reaction mixture was made with 40 µL of sucrose (480 mM), 10 µL of enzymatic extract dilutions and 50 µL of PBS. Mixtures were incubated for an hour at 37°C, followed by 10 minutes at 100°C to finish the reaction. Free glucose concentration was determined using Spinreact kit (Girona, Spain).

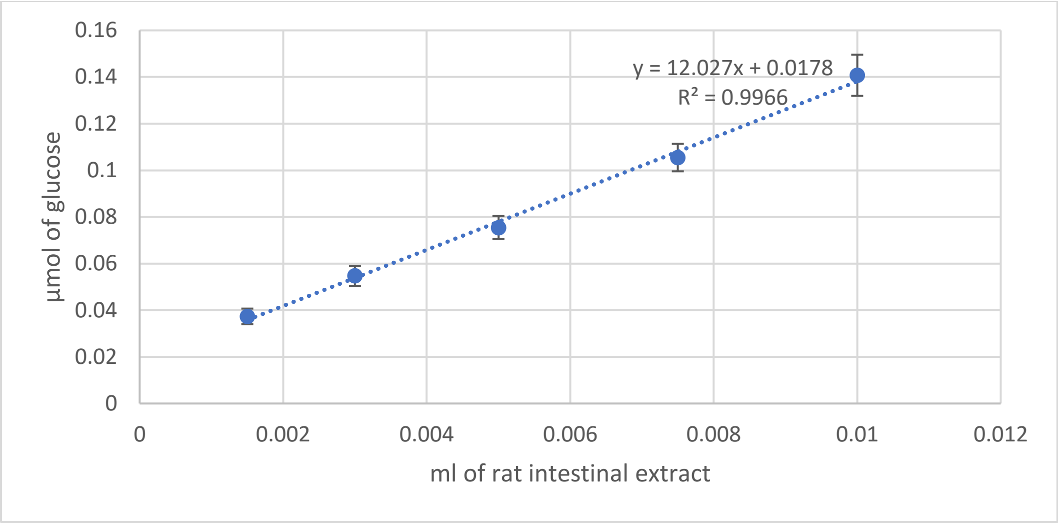

Figure S7- Glucose released from sucrose due to sucrose activity of rat intestinal extract

Sucrose activity of rat intestinal extract was of 0.254 ± 0.028 µmol of glucose
